# Supplementary material for: Controlling Legionella pneumophila in Showerheads: Combination of Remedial Intervention and Preventative Flushing
Source: Microorganisms. 2023 May 23;11(6):1361. doi: 10.3390/microorganisms11061361 (PMC10301046; doi:10.3390/microorganisms11061361)
Supplement: Supplementary file 1 [file microorganisms-11-01361-s001.zip › microorganisms-2402433-supplementary.pdf]

microorganisms

MDPI

Supplementary Materials

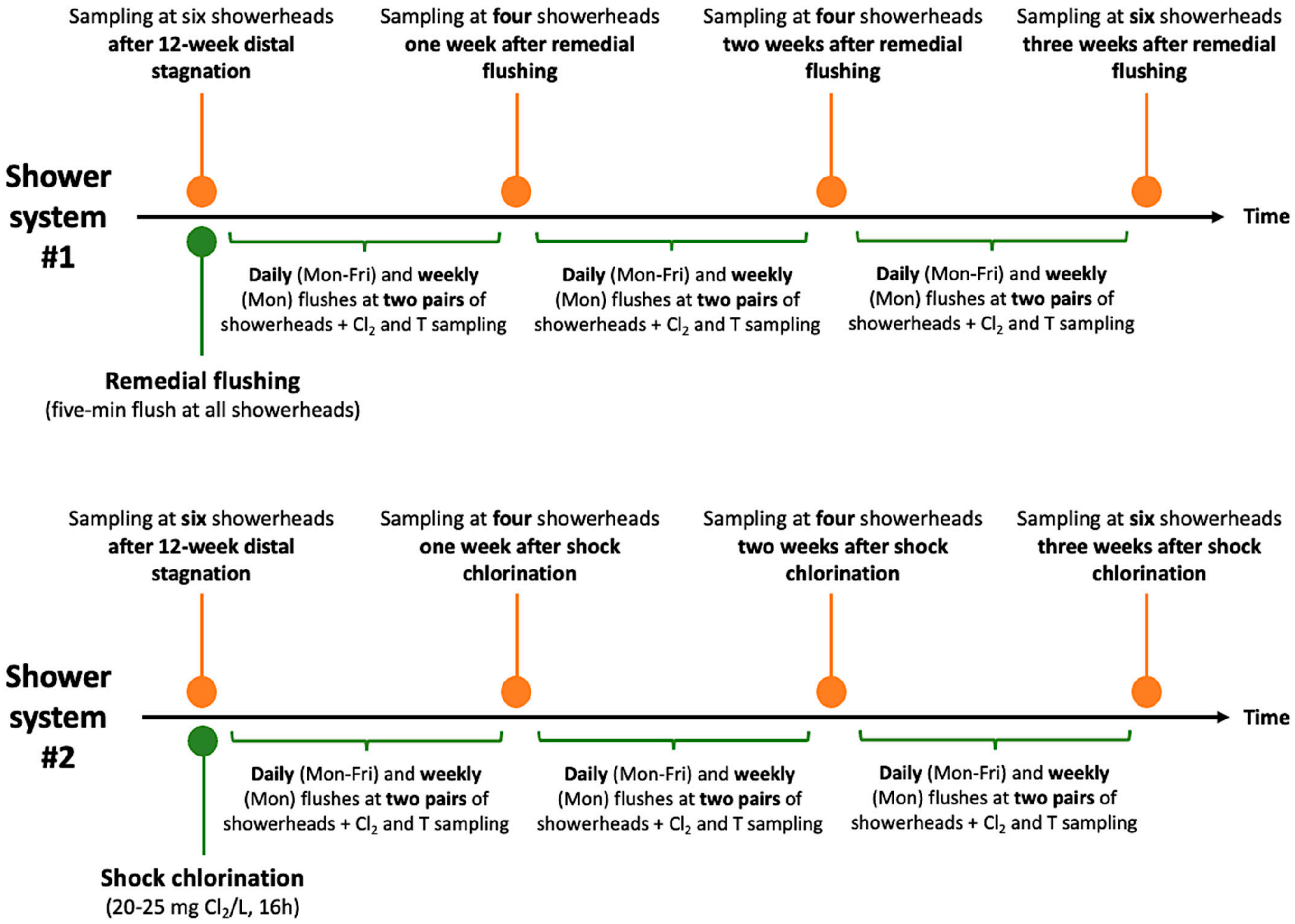

Figure S1. Chronological steps of the sampling events (orange) and interventions (green) carried out in both shower systems.
